# Sirenomelia: A Review of European Prevalence Data and Epidemiological Analysis of 17 Cases Registered in Wales

**DOI:** 10.1002/bdr2.70051

**Published:** 2026-05-06

**Authors:** Chris Emmerson, Michael Olson, Ceri Williams, Robert Maddison, David Tucker, Llion Davies, Annie Perraud, Linda Bailey, Ciarán Humphreys, Rhian Hughes, Joanna Sichitiu, Madalen Oribe Amores, Ingeborg Barisic, Michele Santoro, Clara Cavero Carbonell, Elizabeth S. Draper, Martin Haeusler, Isabelle Monier, Anna Latos‐Bielenska, Carlos Matias Dias, Elly Den Hond, Elisa Ballardini, Mary O'Mahony, Isabelle Perthus, Judith Rankin, Anke Rissmann, Florence Rouget, Sarah Stevens, Jorieke E. H. Bergman, Diana Wellesley, Wladimir Wertelecki

**Affiliations:** ^1^ Public Health Wales Cardiff UK; ^2^ Wales Gene Park, Wales Genomic Health Centre Cardiff University Cardiff UK; ^3^ Public Health Wales Singleton Hospital Swansea UK; ^4^ European Commission, Joint Research Centre (JRC) Ispra Italy; ^5^ Public Health Wales Carmarthen UK; ^6^ Department of Woman‐Mother‐Child University Medical Center CHUV Lausanne Switzerland; ^7^ Registro Anomalías Congénitas CAV, Subdirección de Salud Pública Bilbao Spain; ^8^ Children's Hospital Zagreb Medical School University of Zagreb Zagreb Croatia; ^9^ CNR Institute of Clinical Physiology Pisa Italy; ^10^ Fundación para el Fomento de la Investigación Sanitaria y Biomédica de la Comunitat Valenciana (FISABIO), Centro Superior de Investigación en Salud Pública Valencia Spain; ^11^ Department of Population Health Sciences The University of Leicester Leicester UK; ^12^ Styrian Malformation Registry Medical University of Graz Graz Austria; ^13^ INSERM U1153, Obstetrical, Perinatal and Pediatric Epidemiology Research Team (EPOPé), Center of Research in Epidemiology and Statistics, Sorbonne Paris Cité Paris France; ^14^ Department of Medical Genetics Polish Registry of Congenital Malformations Poznan Poland; ^15^ Centro de Estudos e registo de A C Lisbon Portugal; ^16^ Provinciaal Instituut voor Hygiene Antwerp Belgium; ^17^ IMER Registry (Emilia Romagna Registry of Birth Defects), Centre for Clinical and Epidemiological Research University of Ferrara and Azienda Ospedaliero Univerisitarion di Ferrara Ferrara Italy; ^18^ Department of Public Health HSE South (Cork & Kerry), St. Finbarr's Hospital Cork Ireland; ^19^ CEMC—Auvergne Chamalieres Cedex France; ^20^ Institute of Health & Society Newcastle University Newcastle upon Tyne UK; ^21^ Malformation Monitoring Centre Saxony‐Anhalt, Medical Faculty Otto‐von‐Guericke University Magdeburg Germany; ^22^ Brittany Registry of Congenital Malformations, CHU Rennes, Univ Rennes, Inserm, EHESP, Irset (Institut de recherche en santé, environnement et travail)—UMR_S 1085, F‐35000 Rennes France; ^23^ National Congenital Anomaly and Rare Disease Registration Service (NCARDRS), Public Health England London UK; ^24^ Department of Genetics University of Groningen, University Medical Center Groningen Groningen the Netherlands; ^25^ Wessex Clinical Genetics Service, Faculty of Medicine University Hospitals Southampton Southampton UK; ^26^ OMNI‐Net for Children Rivne Ukraine

**Keywords:** congenital abnormalities, mermaid syndrome, sirenomelia, Wales

## Abstract

**Background:**

Sirenomelia is a rare congenital condition most notably characterized by a single lower limb. Previous studies have suggested a prevalence of approximately 1 per 100,000 births. However, in Wales 17 cases were recorded between 1998 and 2016, suggesting a higher rate of sirenomelia in this country.

**Objectives:**

This study compared current prevalence of sirenomelia in Wales with European data. This study further reviewed detailed time, place, and person data on sirenomelia cases in Wales to investigate possible causal factors.

**Method:**

A retrospective cohort study from birth defect surveillance programs. Individual‐level records for all welsh cases were examined for evidence of causal factors. Comparator data from other countries were obtained from EUROCAT (a European network of population‐based registries for the epidemiological surveillance of congenital anomalies).

**Results:**

European data from 24 national and regional registries of congenital anomalies included 97 cases of sirenomelia identified across 9.6 million births between 1998 and 2016, giving a prevalence rate of 1 per 100,000 (95% CI 0.83, 1.23). Five regions reported statistically significantly higher rates than that recorded across all registries, while one region reported a significantly lower rate. Small numbers of cases limit definitive statistical interpretation. Analysis of the Wales data did not identify common epidemiological factors between cases.

**Conclusions:**

The contemporary prevalence of sirenomelia across Europe is consistent with earlier studies at approximately 1 per 100,000 births (95% CI 0.83, 1.23). However, there is greater variation between regions than would be expected by chance. There remains no definitive evidence for causal environmental factors.

## 1 Background

Sirenomelia is a rare congenital condition characterized by malformations of the urogenital and lower gastrointestinal tract, defects in the lower spinal column and a single lower limb (Cozzolino et al. 2016). The etiology of sirenomelia is not well understood. Sirenomelia was previously thought to be a severe form of caudal regression syndrome, but it has been disputed that they are separate entities with similar origins (Isik Kaygusuz et al. 2016). There are two common hypotheses for sirenomelia. The “vascular steal” hypothesis suggests a vitelline blood vessel develops which results in inadequate blood flow to the lower part of the fetus, leading to underdeveloped organs and the development of the single lower limb (Isik Kaygusuz et al. 2016). The “blastogenesis” hypothesis suggests sirenomelia is caused by disruption to the development of the caudal mesoderm (Castori et al. 2010). These hypotheses do not exclude each other.

Large‐scale studies of records held on national registers of congenital anomalies suggest a total prevalence of approximately 1 case per 100,000 births (Orioli et al. 2011). In Wales, the Congenital Anomaly Register and Information Service (CARIS) recorded 17 cases between 1998 and 2016. With a total of 635,876 births recorded in Wales in that period (Office for National Statistics 2011), this suggests a total birth prevalence rate of approximately 2.7 cases of sirenomelia per 100,000 in Wales over this period (95% CI 1.7–4.3). This rate was higher than would be expected given previous epidemiological studies of sirenomelia.

The aims of this study were to compare the rates of sirenomelia in Wales with other regions and investigate potential factors that may be associated with clusters of cases.

## 2 Methods

### 2.1 Prevalence Data

The European Concerted Action on Congenital Anomalies and Twins (EUROCAT) is an organization that runs a network of congenital anomaly registries in 39 regions across 21 European countries. Data were requested from EUROCAT for all cases coded as Q87.24 (ICD‐10 British Paediatric Association supplement) and 759.844 (ICD‐9) for all periods and all registries. All cases of sirenomelia were manually validated to ensure no misclassification, such as inclusion of caudal regression cases. Single‐year figures would typically be too low to make assumptions regarding trends over time. Therefore, cases, births and rates were provided for the total period for each registry, as well as for aggregated three‐year periods. Rates and upper and lower 95% confidence intervals were calculated using the Wilson method (Altman et al. 2000). EUROCAT definitions for sirenomelia do not include fetal losses earlier than 20 weeks. Therefore, three cases recorded on CARIS that met this definition were excluded from the analysis.

### 2.2 Wales Case Data

Data were obtained from the Congenital Anomaly Registration & Information Service (CARIS, a population‐based surveillance registry hosted by the National Health Service), on cases of sirenomelia registered between 1998 and 2016. A scoping review of peer‐reviewed literature was carried out using Medline and Embase. The review aimed to identify papers that presented evidence for causal factors associated with sirenomelia based on reported cases (rather than descriptions of theoretical biological or genetic mechanisms). After reviewing the literature, several factors were considered to possibly increase the risk of sirenomelia, including genetics, environment, and maternal health. Descriptive statistics were reported. Given the relatively low numbers of cases, formal statistical tests of comparison were not undertaken.

## 3 Results

### 3.1 Prevalence Data

**FIGURE 1:**
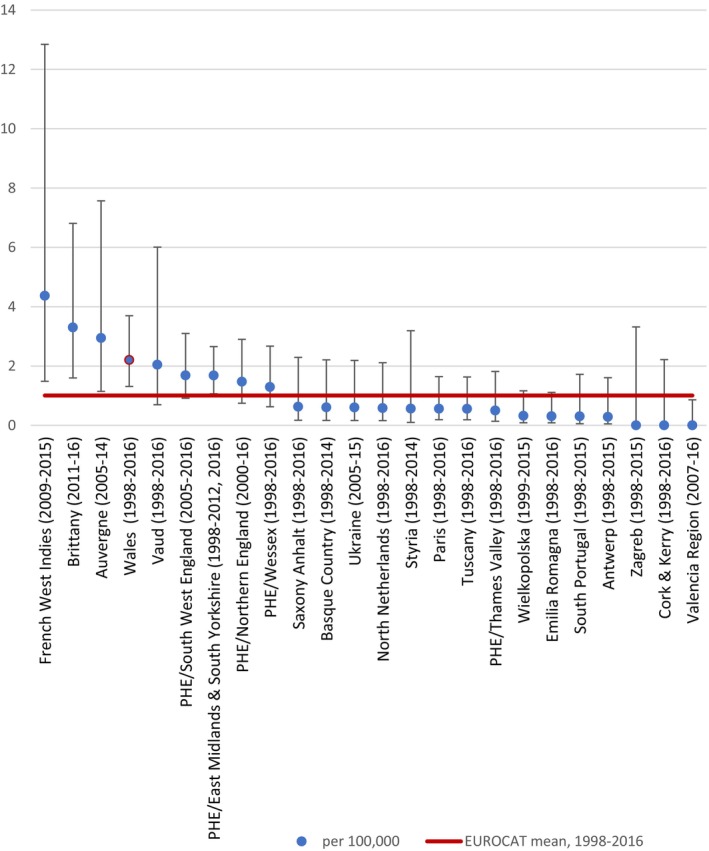
Rates of sirenomelia per 100,000 births and 95% confidence intervals by EUROCAT region and period.

**FIGURE 2:**
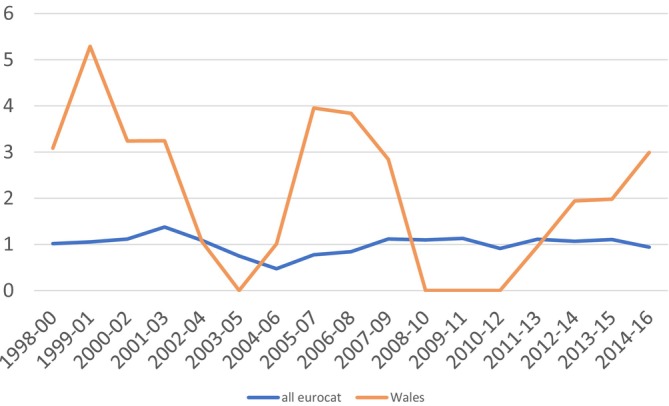
Rates of sirenomelia per 100,000 births, 1998–2016, Wales and all EUROCAT registries.

Data provided by EUROCAT are shown by region for the entirety of birth years for which they have data available in Table 1 and Figure 1. Rates for Wales and for all EUROCAT registries by aggregated 3‐year periods are shown in Figure 2. There were 97 cases (excluding fetal losses at less than 20 weeks) recorded on EUROCAT registries over the period 1998 to 2016 among 9,637,494 births. The rate of sirenomelia per 100,000 births was 1.01 (95% CI 0.83, 1.23).

**TABLE 1:** Cases of sirenomelia, number of births, rates per 100,000 births, and 95% confidence intervals by EUROCAT region and period.

Region	Years	Cases	Births	Cases per 100,000 births	95% CI
Zagreb (Croatia)	1998–2015	0	115,785	0.00	0–3.32
Cork and Kerry (Ireland)	1998–2016	0	173,269	0.00	0–2.22
Valencia Region (Spain)	2007–2016	0	446,903	0.00	0–0.86
Antwerp (Belgium)	1998–2015	1	352,745	0.28	0.05–1.61
South Portugal	1998–2015	1	329,583	0.30	0.05–1.72
Emilia Romagna (Italy)	1998–2016	2	656,668	0.30	0.08–1.11
Wielkopolska (Poland)	1999–2015	2	626,876	0.32	0.09–1.16
Thames Valley (England)	1998–2016	2	401,547	0.50	0.14–1.82
Tuscany (Italy)	1998–2016	3	541,260	0.55	0.19–1.63
Paris (France)	1998–2016	3	536,936	0.56	0.19–1.64
Styria (Austria)	1998–2014	1	177,575	0.56	0.1–3.19
North Netherlands	1998–2016	2	345,475	0.58	0.16–2.11
Ukraine	2005–2015	2	333,189	0.60	0.16–2.19
Basque Country (Spain)	1998–2014	2	330,187	0.61	0.17–2.21
Saxony Anhalt (Germany)	1998–2016	2	318,270	0.63	0.17–2.29
Wessex (England)	1998–2016	7	541,068	1.29	0.63–2.67
Northern England	2000–2016	8	544,479	1.47	0.74–2.9
East Midlands and South Yorkshire (England)	1998–2012, 2016	18	1,071,924	1.68	1.06–2.65
South West England	2005–2016	10	594,306	1.68	0.91–3.1
Vaud (Switzerland)	1998–2016	3	146,794	2.04	0.7–6.01
Wales	1998–2016	14	635,876	2.20	1.31–3.7
Auvergne (France)	2005–2014	4	135,908	2.94	1.14–7.57
Brittany (France)	2011–2016	7	212,206	3.30	1.6–6.81
French West Indies	2009–2015	3	68,665	4.37	1.49–12.85
Total (all registries, all years)		97	9,637,494	1.01	0.83–1.23

In five regions, the reported rates were significantly higher: French West Indies (4.37, 95% CI 1.49, 12.85), Brittany (3.3, 95% CI 1.6, 6.81), Auvergne (2.94, 95% CI 1.14, 7.57), Wales (2.2, 95% CI 1.31, 3.7), and the East Midlands and South Yorkshire (EMSY, 1.68, 95% CI 1.06, 2.65). In Valencia, the rate was significantly lower (0, 0–0.86).

Wales and EMSY recorded peak 3‐year rates in 1999–2001 (5.3 per 100,000) and 2009–11 (3.1), respectively, and peaks in Auvergne and Brittany were recorded in 2012–2014 (7.5) and 2014–2016 (3.9). The peak value across EUROCAT registries was 1.4 cases per 100,000, recorded in 2001–03. The lowest rate recorded across all EUROCAT registries was 0.5 per 100,000 in 2004–2006. These overall rates have been notably stable in recent years, with the rate remaining within the range of 0.9 to 1.1 in every period between 2007–2009 and 2014–2016.

### 3.2 Wales Case Data

An analysis of Wales data obtained from CARIS reported 17 cases of sirenomelia between 1998 and 2016. A postmortem was known to have been performed for 13 of the 17 cases (76.5%), although the result was not known in one case.

Data available on CARIS included full postcodes, thus allowing cases to be identified with a high level of geographical precision (often to street level). Of the 17 cases that CARIS registered between 1998 and 2016, three were recorded in the Betsi Cadwaladr University Health Board (one of Wales's seven local health boards, serving the population of North Wales), three in Cardiff (Wales's capital city and largest population center), three across the area of the Swansea Bay and Hywel Dda University Health Boards, and eight across the area of South Wales included within the Cwm Taf and Aneurin Bevan University Health Boards (historically these areas contained industry such as coal mining and steel). There were four cases recorded in 2007, three in 1999 and two in both 2001 and 2016. There was one case recorded in each of 2000, 2002, 2004, 2006, 2013, and 2014.

No evidence was found to support any geographical or time‐based link between cases given these observed distributions in place and time.

Maternal age range was 15–40, with a mean of 28 years and 3 months and a median of 28 years. In two cases, the women were under 21 and in a further two they were 35 or older. One was a migrant, having moved into Wales from another country.

Four women were coded as having obesity (body mass index = > 30) prior to pregnancy and three were recorded as having asthma, with one also recorded as having type 1 diabetes. There was one case with reported thalassemia, one recorded as having a mental health condition, and one with a history of trophoblastic disease.

Seven women (41.2%) were recorded as having taken folic acid. The only other medications or supplements recorded were antibiotics (one case), over‐the‐counter painkillers like paracetamol, and four cases of medication/supplements appropriate for recorded conditions or pregnancy (such as insulin and ferrous sulfate).

Six women (35.3%) were recorded as smokers, split evenly between those smoking 10 or more cigarettes per day and those smoking fewer than 10. No cases of alcohol or drug misuse were recorded.

Maternal occupation was recorded for 11 cases (64.7%). One individual was recorded as working in a factory. All other recorded occupations (e.g., “housewife,” “support worker,” “teacher”) can be assumed not to involve contact with harmful materials.

There was one case of consanguinity, although the relationship was not recorded.

#### 3.2.1 Characteristics of Fetus, Pregnancy, and Birth

The gender of the fetus was recorded as male in seven cases and female in eight cases, with gender not recorded in the remaining cases. There was one case in which a different congenital anomaly had been recorded for a previous child. Additional anomalies were recorded for 16 of the 17 cases (94.1%). A total of 133 additional anomalies were recorded, equating to a mean of 7.8 per case. The highest number of additional anomalies recorded for a single case was 14. Anomalies were consistent with descriptions of sirenomelia in the literature, with the most frequently recorded anomalies being the absence, agenesis, hypoplasia or dysplasia of organs (70 instances, 52.6% of all anomalies recorded). The next most common groups of additional anomalies were spine or limb fusion (16 instances, 12%) and anomalies related to artery formation (15 instances, 11.3%). One instance of twinning was recorded. Only one identified case of sirenomelia ended in a birth, with the baby recorded as dying on the same day.

## 4 Discussion

The total birth prevalence rate of sirenomelia in Wales was approximately 2.7 cases per 100,000 births (95% CI 1.7–4.3). Despite being significantly higher than the EUROCAT average (1.01, 95% CI 0.83, 1.23), other registries contributing data to EUROCAT also reported prevalence rates significantly higher than the average. From the broader global literature, geographical variation in prevalence rates was noted. There were contrasts between data collected by EUROCAT registries and those presented by Orioli et al. (2011). Three regions in the analysis by Orioli et al. (2011) recorded rates significantly higher than the rate for all registries (0.98 per 100,000), of which two (South American ECLAMC, 1.36 per 100,000, 95% CI 1.04, 1.74 and Mexico RYVEMCE, 2.36, 95% CI 1.53, 3.49) were outside of Europe and therefore not included in EUROCAT figures. Northeast Italy also recorded a significantly higher rate (1.69, 95% CI 1.03, 2.60), but the Campania region recorded a significantly lower rate (0.16, 95% CI 0.00, 0.87), as did Hungary (0.33, 95% CI 0.16, 0.61). No Hungarian region was included in the EUROCAT data. There are, therefore, no clear geographical trends in the periods covered by the two analyses.

Another posited theory involves fallout from the 1986 Chernobyl disaster in Ukraine. In subsequent months, heavy rain brought higher quantities of radioactive cesium and iodine to the counties of Cumbria, Clwyd, and Gwynedd (Howard and Beresford 1989). However, no correlation between fallout from the accident and congenital anomaly prevalence rates in Wales or England. Moreover, Bentham (1991) concluded there is “no evidence that radiation from Chernobyl caused a rise in perinatal mortality in England and Wales.”

After reviewing the literature, geographical and several maternal factors were considered as potential causes of the Wales sirenomelia cluster. However, no explanation was identified for the high sirenomelia prevalence in Wales between 1998 and 2016.

Recent animal studies suggest that the etiology of sirenomelia may have a genetic component. Mice lacking an enzyme that degrades retinoic acid or a protein involved in signaling development of the caudal body develop sirenomelia (Garrido‐Allepuz et al. 2012). This suggests a possible genetic origin. Gabriele and Gianpaolo report evidence of balanced chromosomal translocation in a mother carrying a fetus with sirenomelia (Gabriele and Gianpaolo 2013) and recent research has identified possible pathways by which genetic mutation can lead to sirenomelia in mice (Pennimpede et al. 2010). A de novo balanced 46,X,t(X;16) translocation was reported in a fetus with sirenomelia assigned on chromosomes x and 16 (Kurosawa et al. 2012), while a fetus with mosaic triploidy (69,XXX/46,XX) and sirenomelia was described by Gabriele and Gianpaola in 2013. The risk of sirenomelia has been reported as 100–150 times greater for monozygotic (identical) twins than for dizygotic twins or singleton pregnancies (Boer et al. 2017). However, while studies by Mohamud et al. (2022) and Agrawal et al. (2023) provide examples of cases of sirenomelia in monozygotic twins, incidence in twins is still considered low (Yaşar et al. 2022).

Epidemiological studies of sirenomelia clusters, notably in Cali, Colombia, suggest possible environmental origins for sirenomelia. In 2004–2005, four cases of sirenomelia were recorded over 55 days in Cali, a city with approximately 30,000 births per year (Castilla et al. 2008). This likely cluster has been described and studied by a number of researchers, with proximity to a poorly managed landfill site being the most consistently found factor connecting the cases (Orioli et al. 2009; Saldarriaga et al. 2015). However, evidence for a causal link has been limited and a follow‐up 8 years later noted no further cases (Saldarriaga et al. 2014). While these papers noted hypotheses regarding environmental causes, no confirmed environmental exposures have been identified.

Other causes of sirenomelia have been proposed, including maternal diabetes (Yaşar et al. 2022), use of cocaine (Sarpong and Headings 1992), and exposure to various prescribed medicines, including anti‐epileptics (Tica et al. 2013) and vitamin A (Von Lennep et al. 1985). However, these hypotheses have typically been presented as case studies, with their conclusions not supported consistently across studies and/or failing to offer clear causal hypotheses to link exposures to prevalence of sirenomelia. In contrast, Orioli et al. analyzed data on 25,290,172 births submitted to the International Clearinghouse for Birth Defects Surveillance and Research (ICBDSR) by 19 national or regional surveillance programs to explore factors related to individual rather than environmental causes. This analysis identified 249 sirenomelia cases, with maternal age under 20 identified as the only characteristic that showed a statistically significant association with sirenomelia (35 cases, prevalence ratio 1.71, 95% CI 1.13, 2.59 compared with reference maternal age of 25–29) (Castori et al. 2010).

### 4.1 Strengths and Limitations

The strength of this study derived from the combined methods that considered the European data and an in‐depth review of the Wales registry data. This was deemed a pragmatic approach to explore the evidence in relation to this rare disease. The data available via EUROCAT were standardized according to EUROCAT protocols, thus allowing best comparisons. Additionally, the local Wales data were collected within a long‐established surveillance registry with robust processes and high data quality and capture, meaning that unregistered cases in Wales are unlikely.

However, we acknowledge some limitations. First, the rarity of sirenomelia makes it difficult to identify clusters and to isolate causal factors in the event of an identified cluster. Second, the low numbers of cases of sirenomelia, combined with a dearth of genetic and maternal data within EUROCAT, limited the study's ability to identify definitive evidence for causal environmental factors. Third, it is difficult to rule out chance or artifact (such as differences in coding regimes between geographies) as explanations for the higher‐than‐expected number of sirenomelia cases in Wales over the past two decades, even if some cases were linked by a common factor.

Despite the inclusion of two women of low maternal age and another with type 1 diabetes in the CARIS study, the numbers were too small to permit statistical associations of sirenomelia with potential causes.

## 5 Conclusion

This analysis has not identified any evidence to support a link between environmental factors and the occurrence of sirenomelia. Across Europe, rates of sirenomelia appear stable, with no significant variation over the past 20 years and no long‐term changes observed when comparing contemporary with historical data. There was considerable variation in rates between regions and time periods. However, these variations typically involve small numbers of cases, and no common themes emerged from the analysis that would suggest factors common to different times and places. The nature of sirenomelia, particularly its rarity, may result in differences in recording between and within registries, and therefore caution should be exercised in interpreting variations in case numbers and rates. Continued monitoring of cases by national registries may enable further analyses with a greater number of registered cases in the future.

## Funding

The authors have nothing to report.

## Ethics Statement

The authors have nothing to report.

## Consent

The authors have nothing to report.

## Conflicts of Interest

The authors declare no conflicts of interest.

## Data Availability

The data used in this study were securely provided by EUROCAT and CARIS, Public Health Wales. Requests for de‐identified data should be directed to these organizations.
